# Can Treatment With Citicoline Eyedrops Reduce Progression in Glaucoma? The Results of a Randomized Placebo-controlled Clinical Trial

**DOI:** 10.1097/IJG.0000000000001565

**Published:** 2020-06-11

**Authors:** Luca Rossetti, Michele Iester, Laura Tranchina, Laura Ottobelli, Giulia Coco, Elisabetta Calcatelli, Chiara Ancona, Paola Cirafici, Gianluca Manni

**Affiliations:** * Eye Clinic, ASST Santi Paolo e Carlo, University of Milan, Milan; † Eye Clinic, DiNOGMI, University of Genoa; ‡ IRCCS Policlinico San Martino Hospital, Genova; § Department of Clinical Sciences and Translational Medicine, University of Tor Vergata; ∥ Bietti Foundation, IRCCS, Rome, Italy

**Keywords:** glaucoma, neuroprotection, clinical trial, citicoline

## Abstract

**Precis:**

Citicoline eyedrops in patients with progressing glaucoma.

**Purpose:**

This study aimed to test whether the additional therapy with citicoline eyedrops to intraocular pressure (IOP)-lowering treatment could slow glaucoma progression in patients with worsening of damage and IOP 18 mm Hg or less.

**Design:**

This was a randomized, double-masked, placebo-controlled, multicenter 3-year study.

**Outcomes:**

The outcomes studied were difference in the visual field (mean deviation, MD, of 24-2; MD of 10-2) rates of progression and difference in retinal nerve fiber layer (RNFL) thickness change between the 2 study groups at 3 years.

**Methods:**

Patients with mild to moderate open-angle glaucoma (OAG) showing damage progression of at least −0.5 dB/y in the 2 years before enrollment despite IOP ≤18 mm Hg were randomized to receive citicoline eyedrops or placebo 3 times daily for 3 years. Patients were followed every 3 months and underwent a visual field examination with 24-2 and 10-2 strategies and RNFL assessment. Analysis of variance and linear models were used to test the differences between groups.

**Results:**

Eighty patients were randomized in the trial. The mean 3-year rates of progression were −1.03 (2.14) dB in the citicoline group and −1.92 (2.23) dB in the placebo group (*P*=0.07) for 24-2 MD and −0.41 (3.45) dB in the citicoline group and −2.22 (3.63) dB in the placebo group (*P*=0.02) for 10-2 MD. On average, patients receiving citicoline eyedrops lost 1.86 μm of RNFL in 3 years, versus 2.99 μm in the placebo group (*P*=0.02).

**Conclusions:**

Additional treatment with citicoline eyedrops to IOP-lowering treatment might reduce disease progression in patients with progressing glaucoma despite IOP ≤18 mm Hg.

Open-angle glaucoma (OAG) is a progressive optic neuropathy where intraocular pressure (IOP) plays a major role of the main and successfully treatable risk factor.^1^ Today, there is plenty of evidence indicating that IOP-lowering can have a huge effect on disease progression.^2^ However, clinical trials and “every-day” clinical experience clearly show that, despite “apparently good” IOP values, glaucoma damage can significantly increase in a number of patients, suggesting that the neurodegeneration might have an IOP-independent pathway.^3^

The concept of neuroprotection in glaucoma was first introduced more than 15 years ago^4^ and since then, a number of molecules potentially acting as “neuroprotectors” have been tried in experimental studies, with very interesting findings.^5^ However, despite the bulk of experimental evidence, the clinical results of neuroprotection in human glaucoma have never been conclusive. A meta-analysis of clinical trials on neuroprotection in glaucoma reported a substantial lack of evidence.^6^

Among various molecules with a putative role in reducing neurodegeneration, citicoline has been found to have a “clinically significant” effect in many neurodegenerative diseases, including senile dementia and stroke.^7^ Citicoline could have various mechanisms of action including the preservation of cardiolipin and sphingomyelin, restoration of phosphatidylcholine, stimulation of glutathione synthesis, lowering glutamate concentration, rescuing mitochondrial function, and others.^8^ The potential action of citicoline in ameliorating glaucoma neurodegeneration has been studied for many years with promising findings.^8^–^14^

In the first studies on glaucoma, citicoline was administered through intramuscular injections as for other neurodegenerative diseases. Needless to say that this way of administration was rather unpractical for glaucoma patients.^11^,^12^ In more recent years, other ways of administrations (ie, oral solution and eyedrops) were proposed, with encouraging results.^9^,^10^,^13^ Unfortunately, reports of observational studies or undersized trials, although showing interesting findings, did not provide conclusive evidence. As an example, Parisi and colleagues recently found that treatment with topical citicoline was able to enhance the bioelectrical responses [pattern electroretinogram (PERG) amplitude] and improve the bioelectrical activity of the visual cortex [visual evoked potential (VEP) implicit time and amplitude] in patients with mild glaucoma and IOP 18 mm Hg or less under medical treatment. Although statistically significant results on^12^ patients at 4 months were reported, no conclusion could be drawn about the effect of citicoline on vision as long-term data on visual field (VF) changes were not shown.^9^

The aim of the present trial was to assess whether additional treatment with topical citicoline could have an effect on disease progression in OAG patients showing progressing damage despite IOP≤18 mm Hg.

## METHODS

The trial was conducted at 3 University Eye Clinics in Milan, Rome, and Genoa between summer 2015 and spring 2019. The study was designed following the tenets of the Declaration of Helsinki and the protocol was submitted and approved by each University Ethics Committee. The trial was funded by the “Istituto di Ricerca in Neuroftalmologia” (I.R.N.) s.r.l., Rome, Italy, and by Omikron Italia, and registered (NCT04020705, clinicaltrials.gov).

### Patients in the Trial

Patients in the trial were a highly selected sample from 3 large University Eye Clinics practices that follow thousands of glaucoma cases every year. All medical records were carefully inspected to identify potentially eligible cases. In particular, patients had to be followed with a diagnosis of OAG for more than 2 years, had to have at least 2 VF tests per year, and information on IOP from at least 2 recordings per year. This screening was performed through a search in the hospital databases. The patients’ charts were collected and inclusion criteria were verified directly from medical records that report all patients data with VF, ocular coherence tomography (OCT) printouts, tonometric curves, pachymetry, and all ophthalmological findings. All potentially eligible cases were then called for a screening visit. A total of 350 patients underwent an ophthalmic assessment and a VF test; out of these, 270 could not be considered as candidates for trial purposes: 105 cases did not confirm progression rates at VF testing, 151 showed IOP readings above 18 mm Hg, 10 had a mean deviation (MD) values worse than −15 dB, and 4 changed their mind about participation to the study.

Inclusion criteria were as follows: patients least 18 years of age with OAG (primary or pseudoexfoliative) in at least 1 eye; patients who were familiar with VF tests (ie, they had ≥4 tests in the past 2 y) and showed mild to moderate typical and reliable glaucomatous VF loss (MD, between −2 and −15 dB on Humphrey field analyzer, HFA, 24-2 SITA Standard strategy); patients with glaucomatous damage progression in the past 2 years: MD at the screening visit had to be worse of at least – 1 dB than 2 years before (rate of progression of at least −0.5 dB/y); best-corrected visual acuity (BCVA) of 20/40 or better and media opacities not significantly interfering with VF results; and patients with IOP values not higher than 18 mm Hg at least in the last 2 years with any available therapy (ie, medical, SLT or surgical). IOP needed to remain ≤18 mm Hg in the study eye throughout the 3-year trial period; if, during the study, IOP was found to be above 18 mm Hg, a new treatment regimen was offered to control IOP. If the new treatment option was either refused or not effective, the patient was excluded from the study. Patients with other types of glaucoma or supplemented with oral “potential neuroprotectors” (any molecules with a known or supposed neuroprotective effect, eg, Gingko Biloba, Coenzyme Q10, Memantine, Citicoline, etc.) were not eligible. Patients with other diseases (local or systemic, eg, diabetic retinopathy, age-related macular degeneration, ischemic optic neuropathy, pathologies of the visual pathways) that could affect VF test results according to the investigator’s judgment were also excluded.

### Study Design

This study was a randomized, placebo-controlled, double-masked trial. If eligible at the screening visit, patients had to sign the informed consent, a baseline visit was planned, and then they were randomized to receive either citicoline eyedrops or placebo 3 times daily (8 am, 2 pm, and 8 pm) for 3 years. Four visits per year were then planned: each visit included a complete ophthalmologic assessment (with BCVA, refraction, anterior, and posterior segment evaluation, and tonometry) and a VF test. The VF was tested with both 24-2 and 10-2 HFA strategies: at baseline, 6-, 12-, 18-, 24-, 30-, and 36-month visits, a 24-2 strategy was tested, whereas at baseline, 3-, 9-, 15-, 21-, 27-, 33-month visits, a 10-2 strategy was used. Therefore, at the end of the trial, patients completed seven VF tests with both strategies. In addition, optic nerve head and retinal nerve fiber layer (RNFL) OCT were assessed at baseline and every 6 months.

An automated system was used to generate 3 randomization lists (1 per site) and adopted by the central pharmacy to prepare the drug kits for the patients of the 3 centers.

### Study Treatments

Patients received the best possible treatment options to control IOP and maintain visual function. According to the protocol, all necessary treatments (including surgery) to maintain IOP 18 mm Hg or less were offered to the patients during the study. In addition, citicoline eyedrops (OMK1, Omikron, Italy, citicoline sodium salt: 0.2 g, Hyaluronic acid: 0.02 g, Benzalkonium chloride: 0.001 g, water for injection up to 10 mL) and placebo (vehicle, Hyaluronic acid, Benzalkonium chloride: 0.001 g, water for injection up to 10 mL) were prepared in identical bottles to maintain masking conditions for both patients and evaluators. The bottles, labeled with the assigned randomization number, were provided directly to the clinical centers by an independent pharmacy. Study drugs were then kept in the hospital pharmacy store and provided to the patients every 6 months. On this occasion, the patients received the box with the bottles for 6-month therapy and gave back to the pharmacist the bottles with the drugs used during the previous 6-month period. During each visit, the patients were asked about compliance to the study drugs and information was recorded in the “patient’s source document.”

### Study Outcomes

Primary outcomes were the differences in VF progression (MD) rates either assessed with the 24-2 (SITA standard) strategy or with the 10-2 strategy of HFA between the 2 groups. Secondary outcomes were the change in OCT RNFL thickness (average of 4 sectors of Heidelberg Spectralis OCT), and the safety and tolerability of citicoline eyedrops. Although IOP was not a study outcome, in the case of values above 18 mm Hg (at least from 2 consecutive visits), despite changes in IOP-lowering strategies, the patient reached an end-point and was excluded from the study. In the case of discontinuation, patients were still followed, although not part of the study. All IOP values were measured with a calibrated Goldmann tonometer in the morning between 8 and 10 am.

### Analysis

One eye per patient was considered in the analysis. If both eyes were eligible, patients received study treatments in both eyes. In this case, only 1 eye, the one with the worst MD at baseline, was analyzed for study purposes. All analyses were carried out by an independent statistician, unaware of the treatment assignment, using SPSS software (25.0.0.0, 2018). Analysis of variance (ANOVA) and linear models were used to test the differences between groups; the following covariates were tested: age, IOP, MD at baseline, type of diagnosis (primary or pseudoexfoliative), type of therapy (surgery vs. medical therapy), and baseline rates of progression (−0.5 to −0.75 vs. −0.76 to −1 vs. worse than −1 dB/y). Interactions were also tested in the models. Bonferroni’s method was used to “adjust” *P*-values for multiple comparisons. *T* tests were used to compare means at single study intervals.

There are no current data on the potential effect of neuroprotection on glaucoma VF rates of progression. To calculate the study sample size, we started from the baseline rates and “arbitrarily” considered a change of 40% as clinically significant. Thus, assuming a mean 3-year rate of MD (24-2 HFA) change in the placebo group of −2.5 dB (−0.8 dB/y), a mean 3-year rate of MD (24-2 HFA) change in the group receiving citicoline eyedrops of −1.5 dB (−0.5 dB/y), a SD of 1.8 dB, a statistical power of 0.8, and level of significance of 0.05, a total of 80 patients were required.

## RESULTS

A total of 80 patients were randomized (40 received topical citicoline and 40 received placebo). All of the patients completed the 3-year trial, with the exception of 2 (2.5%, both in the placebo arm): in both cases, IOP could not be maintained under 18 mm Hg and patients were excluded from the analysis. Thus, the final analysis was carried out on 78 patients.

The main features of the patients are reported in Table 1. There was no significant difference between the 2 study arms in terms of known main prognostic variables, suggesting that the randomization process was effective in balancing the groups. On average, patients had a diagnosis of OAG for 5 years (range from 30 mo to 11 y). Patients had experience with VF testing and, on average, had undergone 12 examinations (range from 5 to 20 tests). Information about untreated IOP could be obtained for 57 cases (71%): the mean IOP was 24.2 mm Hg (SD=3.8), and in 12 patients, it was 18 mm Hg or less. Patients in the trial were well controlled in terms of IOP: the baseline mean IOP was 14.3 mm Hg in the topical citicoline group and 13.8 mm Hg in the placebo group. To control IOP, more than 2 drugs on average were needed (2.2 and 2.3 in the citicoline and the placebo group, respectively). Patients had moderate glaucoma, with a 24-2 MD around −9 dB and, despite the level of IOP, had been progressing at a rate of −0.8 dB/y in the last 2 years. The ranges of pretrial progression rates are reported in Table 1. Despite good mean BCVA, 28 patients (36%) had a VF paracentral scotoma.

**TABLE 1:**
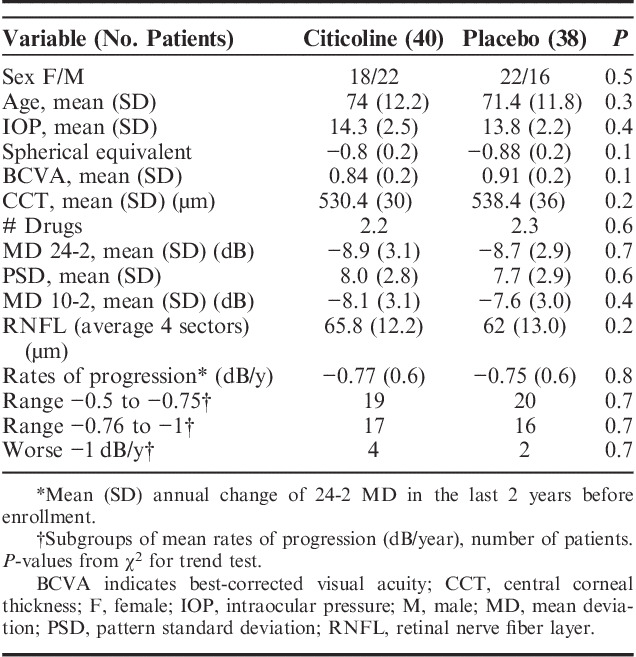
Patients’ Main Characteristics

### IOP in the Trial

**FIGURE 1:**
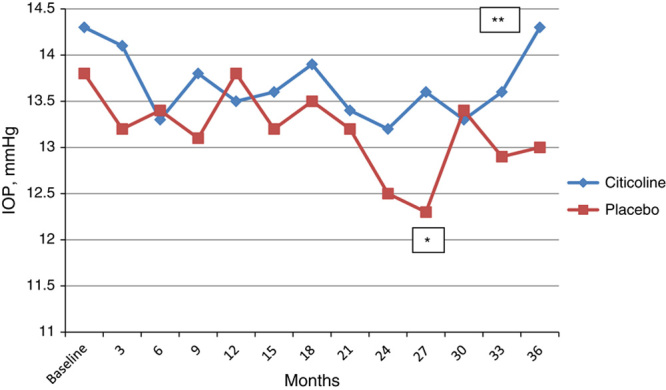
Mean intraocular pressure (IOP) values during the study. *P=0.04; **P=0.05.

Figure 1 shows the IOP profiles during the study. The mean IOP values at different study intervals in the 2 groups are reported in Table 2. The mean pressures ranged from 12.3 to 14.3 mm Hg and were not significantly different from baseline and in the 2 groups; however, at months 27 and 36, IOP was lower in the placebo group than in the citicoline group (13.6 vs. 12.3 mm Hg and 14.3 vs. 13 mm Hg, respectively) and the difference was statistically significant (*P*=0.04 and 0.05, respectively). During the 3-year trial, the IOP-lowering regimen was modified in 25 patients (32%) to control IOP; of these, 3 patients underwent laser trabeculoplasty (1 in the citicoline group and 2 in the placebo group) and 10 underwent trabeculectomy (4 in the citicoline group and 6 in the placebo group). The other 12 patients (7 in the citicoline group and 5 in the placebo group) were shifted from monotherapy to a fixed combination of a prostaglandin and a beta-blocker (3 in the citicoline group and 1 in the placebo group, respectively) and from a fixed combination to a 3-drug regimen (8 patients, 4 in each group).

**TABLE 2:**
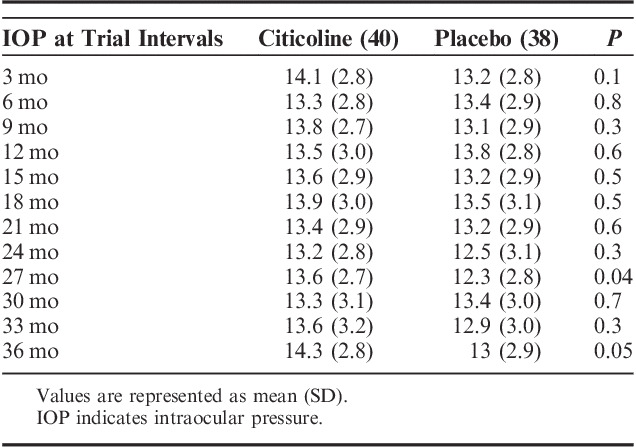
Mean IOP Values in the 2 Study Arms at the Different Trial Intervals

### VFs in the Trial

**FIGURE 2:**
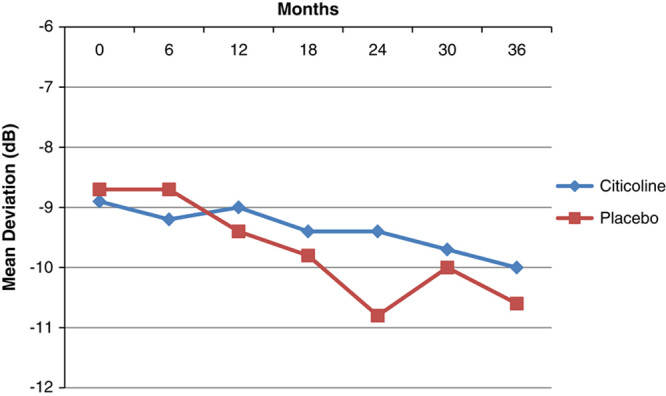
Humphrey field analyzer 24-2 mean deviation changes during the trial in the 2 study arms.

During the study, 30/78 (38%) of the patients progressed according to clinical criteria,^15^ with about one third showing a deepening of the existing scotomas (11 patients) or showing an expansion of scotomas (9 patients) or the combination of the 2 criteria (7 patients), whereas few patients developed a new defect (3 patients). Table 3 reports the mean HFA 24-2 MD values at different study intervals in the 2 groups. During the 3-year trial, the study patients progressed, on average, of −1.46 dB; the 3-year rates of progression were −1.03 dB (2.14) in the citicoline group and −1.92 dB (2.23) in the placebo group (Fig. 2), and, although quite large, the difference did not reach statistical significance (ANOVA, *P*=0.07). Compared with the pretrial rates, both patients receiving citicoline and placebo showed an improvement in the progression rates. At 3 years, there were 3 stable cases (2 in the citicoline group and 1 in the placebo group), 2 of them showing a slight improvement in MD. At baseline, there were just 2 “rapid progressors” (ie, MD change worse than −1.5 dB/y), 1 per treatment arm. In both cases, treatment regimens were changed (1 underwent trabeculectomy) as, during the trial, IOP increased above 18 mm Hg. In both cases, rates of change improved. In the trial, HFA 24-2 MD progression was associated with age (*P*=0.04), but not with IOP (*P*=0.7).

**TABLE 3:**
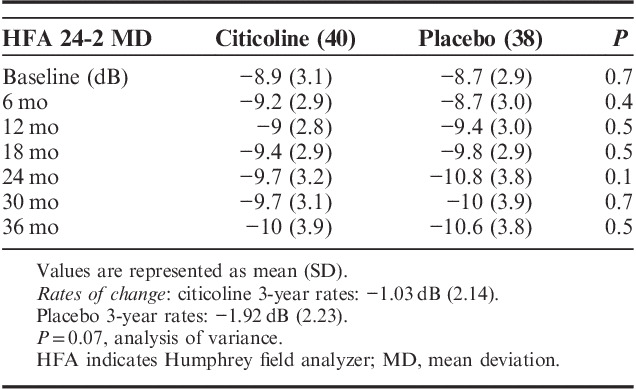
HFA 24-2 MD in the 2 Study Arms at the Different Trial Intervals

**FIGURE 3:**
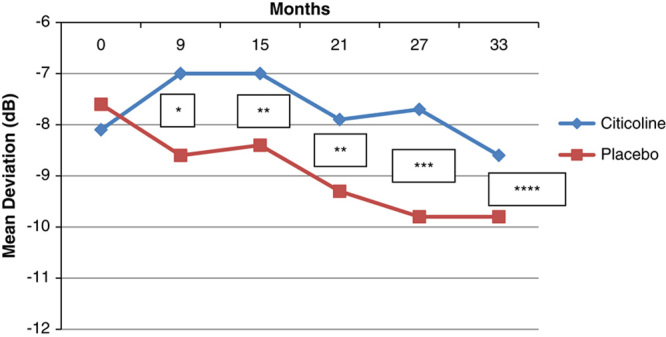
Humphrey field analyzer 10-2 mean deviation changes during the trial in the 2 study arms. *P=0.03; **P=0.04; ***P=0.03; ****P=0.05.

In Table 4, the mean HFA 10-2 MD values at baseline and at different study intervals are presented. With the exception of baseline, MD was always better in citicoline-treated patients than in the placebo group, with the differences being statistically significant at all study intervals. On average, patients receiving citicoline progressed of −0.41 dB in 3 years, whereas patients in the placebo group progressed of −2.22 dB in 3 years (Fig. 3), and the difference was statistically significant (*P*=0.02). A comparison with the pretrial rates was not possible, as patients were not tested with a 10-2 strategy before entering the study. In the model, progression was again associated with age (*P*=0.03) and with the treatment arm (*P*=0.02), but not with IOP (*P*=0.6).

**TABLE 4:**
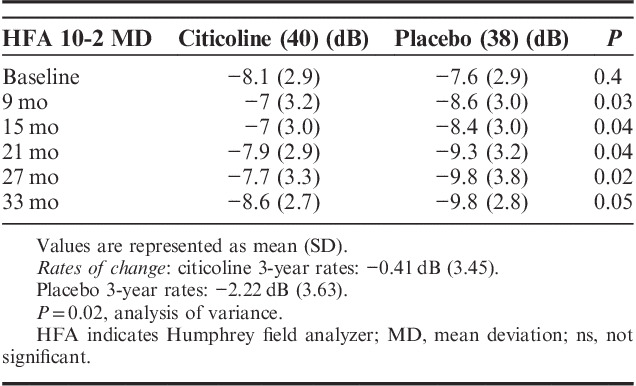
HFA 10-2 MD in the 2 Study Arms at the Different Trial Intervals

### RNFL OCT in the Trial

Patients in the study had moderate glaucoma, with an average RNFL thickness (mean of 4 sectors) of 65.8 μm in the citicoline group and 62 μm in the placebo group. Table 5 shows the mean of RNFL thickness in the 2 groups at the different study intervals. The placebo-treated group had thinner RNFL at baseline and at all study intervals compared with the citicoline-treated group, but the difference was statistically significant only at 30- and 36-month trial points. On average, patients receiving citicoline eyedrops lost 1.86 μm of RNFL in 3 years, versus 2.99 μm in the placebo group (*P*=0.02). Changes in RNFL were associated with changes in 10-2 MD (*P*=0.02), but not with 24-2 MD, age, or IOP. We looked at differences between superior and inferior sectors: citicoline treatment was associated with a reduction of RNFL thinning in the inferior sector (*P*=0.04), compared with placebo, whereas no significant difference could be found for the superior sector (*P*=0.1).

**TABLE 5:**
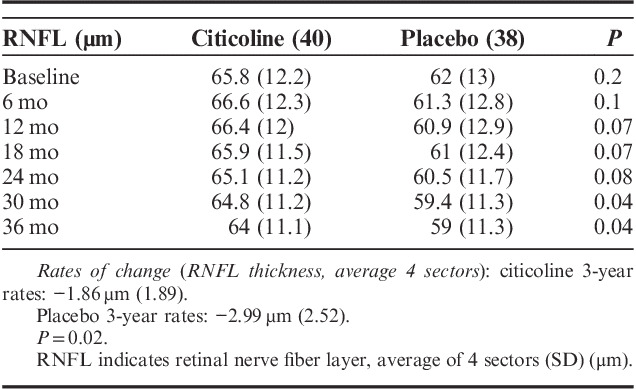
RNFL Thickness in the 2 Study Arms at the Different Trial Intervals

### Subgroup Analyses

The effect of citicoline on 24-2 HFA rates of progression was tested in subgroups of patients in the trial: no significant difference was found in primary OAG versus pseudoexfoliative patients (3-y rates, −1.02 vs. −1.06 dB, *P*=0.3, respectively), in surgically versus medically treated cases (3-y rates, −0.4 vs. −1.1 dB, *P*=0.08, respectively), and in different baseline rates of progression groups (−0.5 to 0.75 dB/y vs. worse than −0.75 dB/y, 3-y rates, −0.6 vs. −1.4 dB, *P*=0.09, respectively). The same comparisons were tested for 10-2 HFA rates of progression and OCT RNFL changes. The mean 3-year rates were −0.39 versus −0.54 dB in OAG versus pesudoexfoliative cases, respectively (*P*=0.8), −0.2 versus −0.43 dB in surgically versus medically treated (*P*=0.7) and −0.31 versus −0.5 dB in slow versus fast progressors at baseline, respectively (*P*=0.8). In terms of RNFL changes, 3-year loss was −1.78 versus −2.42 µm in OAG versus pseudoexfoliative patients, respectively (*P*=0.4), −0.92 versus 1.96 µm in surgically versus medically treated patients, respectively (*P*=0.1), and −1.54 versus −2.14 in slow versus fast progressors at baseline (*P*=0.3).

Citicoline eyedrops were well tolerated and no local or systemic treatment-related side effect was reported during the study. Mild conjunctival hyperemia and ocular surface disease were common problems in the trial reasonably due to the IOP-lowering drugs that the patients were taking. No patient discontinued the study because of ocular or systemic side effects and the trial drugs (ie, citicoline and placebo) did not significantly change the safety profile of the IOP-lowering therapy (Table 6).

**TABLE 6:**
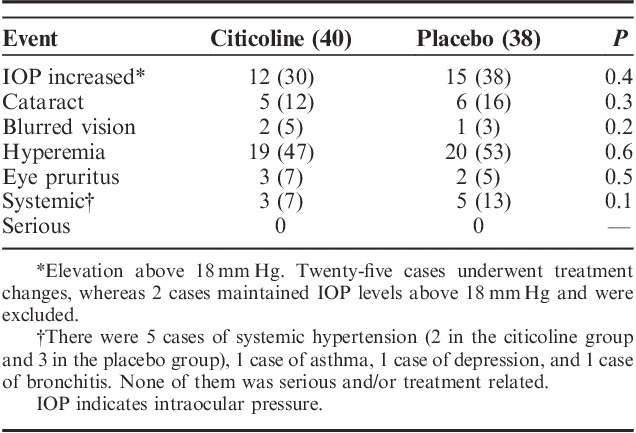
Adverse Events During the Trial (%)

## DISCUSSION

The results of this clinical trial suggest that citicoline eyedrops might slow the disease progression in patients with worsening of glaucoma damage despite IOP ≤18 mm Hg. Although the difference in the rates of progression of HFA 24-2 between the 2 groups did not reach statistical significance, data on progression rates of HFA 10-2 and RNFL OCT showed a statistically significant difference between the citicoline-treated group and the placebo group.

These findings seem to confirm what reported by the copious literature about the effects of citicoline in glaucoma and more in general in neurodegenerative diseases.^7^,^14^ A recent review^8^ extensively summarized the relationship between the cholinergic nervous system and visual function and the potential implications for neuroprotection and/or neuroenhancement in glaucoma. Citicoline (cytidine-5′-diphosphocholine) is a nootropic agent and a central stimulant. It plays an important role in the biosynthesis of phospholipids and their precursors and in maintaining the phospholipid components in the cell membranes. Its mechanism of action is multifactorial and includes (1) preservation of cardiolipin and sphingomyelin; (2) restoration of phosphatidylcholine; (3) stimulation of glutathione synthesis; (4) reduction of glutamate concentration; (5) rescue of mitochondrial function, preventing neural apoptosis; (6) synthesis of myelin; (7) improvements of acetylcholine synthesis; and (8) prevention of endothelial dysfunction. Thus, the neurotherapeutic effect of citicoline could be multifarious, mainly by improving neuronal membrane integrity, maintaining cellular communications with its environment, reducing oxidative stress, and improving the synthesis of neurotransmitters such as acetylcholine and dopamine.^8^ There is, in fact, evidence of a clinical effect of citicoline for a number of neurodegenerative diseases such as Parkinson disease, senile and vascular dementia, and stroke.^16^–^21^

The effect of citicoline on vision has been widely explored. Through the stimulation of the dopaminergic system in the visual pathways, citicoline has been found to improve visual acuity, visual evoked responses, and contrast sensitivity in amblyopia^22^–^24^ and nonarteritic ischemic optic neuropathy.^25^,^26^ In terms of the possible effect of citicoline in glaucomatous neurodegeneration, a number of in vitro and in vivo studies on cell cultures and experimental animal models showed interesting findings of citicoline in regenerating neurites,^27^ protecting from glutamate excitotoxicity,^28^ reducing retinal ganglion cells loss in a crush model^29^ without any significant effect on intraocular pressure,^8^ thus providing direct evidence of neuroprotection. Several clinical studies have shown promising, although not conclusive, results of citicoline treatment in glaucoma patients. Parisi, in a placebo-controlled trial on citicoline administered by intramuscular injections (1000 mg/d), reported improved VEP and PERG, showing that citicoline could ameliorate retinal and bioelectrical responses in glaucoma patients. Results were reported on 40 patients with a follow-up of 6 months and showed that after wash-out, citicoline-related improvements decreased back to baseline values, suggesting that the effect of citicoline might be transient. Unfortunately, no data on VF changes were shown.^12^ A comparison of intramuscular and oral ways of administration found no significant difference in VEP and PERG.^11^ Ottobelli et al,^13^ in an open prospective study, reported that supplementation with citicoline oral solution was associated with a significant change in the rate of progression of VF damage in patients at a high risk of progression of glaucoma (losing at least 1 dB/y of MD) compared with historical controls. The study had several limitations including the design, the small sample size (40 patients), and follow-up duration. More recently, citicoline eyedrops have become available, with potentially better compliance and adherence than oral or intramuscular ways of administration.^30^ Sufficient bioavailability of citicoline at the site of action to the retina was demonstrated in both animal^31^ and human studies.^32^ Parisi et al^12^ could show that the addition of citicoline to IOP-lowering treatment was associated with an improvement in the electrophysiological function of the retina in glaucoma patients. Such an effect was clearly measurable after 4 months of treatment, but regressed to normality after citicoline was stopped during the wash-out, suggesting a neuroenhancer action of the molecule. Unfortunately, this study was not designed to provide a conclusive answer about the effect of citicoline on vision: in particular, the limited sample size (47 patients) and the short duration (6 mo) did not allow for reliable detection of outcomes such as VF changes.

The aim of the present clinical trial was to test whether additional treatment with topical citicoline might have a “clinically significant” effect on glaucoma progression. The study was designed with both functional (primary) and structural (secondary) outcomes. The reason for this choice was to try to differentiate between a neuroenhancement effect, mainly detected by VF changes (improvement?), and a neuroprotective effect that might be assessed through both VF and RNFL OCT changes. In a recent study, Parisi described the effect of citicoline as neuroenhancement (ie, improvements of PEV and ERG) and neuroprotection (ie, improvements of OCT changes) in patients with nonarteritic ischemic optic neuropathy.^25^ In our study, we were able to show a statistically significant difference between citicoline eyedrops and placebo only for HFA 10-2 progression rates, but not for 24-2 rates. There might be different explanations for this result: the higher sensitivity of central 10 degrees VF area in showing changes in trends^33^: the fact that more than one third of the patients had a paracentral scotoma, the lack of statistical power of comparisons, or simply a “chance” effect. In fact, the negative finding about the comparison of 24-2 rates in the 2 groups had only 50% power, due to high variability in observations or limited sample size. The difference in OCT RNFL thickness changes in the 2 groups might suggest a “structural” treatment effect supported by the significant association between 10-2 MD changes and RNFL changes. If this was the case, then citicoline might have shown a “real neuroprotective” action on retinal ganglion cells. Unfortunately, we did not collect the data on OCT macular changes and a better assessment structure/function relationship in these patients could not be possible.

This study has several limitations. First, the trial was probably undersized to reliably detect a “neuroprotective” effect of citicoline. Sample size was calculated on the baseline rates of 24-2 MD change. During the study, rates of progression improved compared with baseline in both study arms, probably because of the better IOP management and the “trial effect.” In the sample size calculation, we underestimated the variability of the progression rates and we did not have sufficient statistical power to provide a conclusive answer about the effect of citicoline on the primary outcome, despite the fact that the observed difference in rates between the 2 arms exceeded 40%. The limited sample size did not allow to draw any conclusions on disease progression using “clinical criteria,” nor on subgroups of patients (pseudoexfoliative glaucomas, surgically treated cases, rapid progressors, etc.). In addition, “trend analyses” of VF changes are probably not sufficient to establish an effect on functional progression of the disease and “event-based” analyses—adopted in major glaucoma clinical trials—should be preferable. A study on neuroprotection in glaucoma has a number of potential confounders, in particular, IOP management during the trial. In the present study, about one third of the patients underwent a change in the therapeutic regimen and ten underwent surgery. It is reasonable to believe that such changes might have played a role in outcome measures, although from a subgroup analysis, surgically treated patients did not show a better outcome than medically treated patients. We attempted to reduce bias by adopting the multicenter, double-masked, placebo-controlled design. The main known prognostic variables, such as age, IOP, number of drugs to control IOP, and glaucoma damage, were equally balanced between the 2 groups at baseline. IOP was well controlled during the trial and, from all analyses, was not found to be associated with VF nor RNFL changes.

In conclusion, the results of this pilot randomized clinical trial might suggest an effect of citicoline in glaucoma, supporting all the literature that, in the last decades, proposed citicoline in neurodegenerative diseases. However, our findings are far from being conclusive for all the study limitations discussed above. Unfortunately, there are still few clinical trials focused on neuroprotection in glaucoma; should our findings be confirmed by larger studies, neuroprotection with citicoline could be proposed as a complementary treatment in the management of patients with “progressing” glaucoma.
